# Examining the effects of upward social mobility on women’s workplace experiences in China

**DOI:** 10.3389/fpsyg.2025.1628896

**Published:** 2025-09-19

**Authors:** Wei Ding, Wen-Cheng Wang

**Affiliations:** The College of Metaverse and New Media, Yango University, Fuzhou, China

**Keywords:** social comparison theory, upward mobility, Chinese women, workplace, thematic analysis model

## Abstract

This study examines how upward social mobility shapes Chinese women’s workplace experiences by applying social comparison theory and asking which structural-related factors most strongly influence women’s perceptions of—and strategies for—occupational advancement in contemporary China. Employing Latent Dirichlet Allocation (LDA) topic modeling on 4,043 Zhihu comments under the “involution” discussion, we identify five operational dimensions—economic reward–value alignment, work burden, institutional environment, resource distribution, and individual agency—that collectively explain variation in perceived mobility opportunities. Our results show that misalignment between compensation and perceived professional worth, coupled with excessive instructional and emotional labor, constitutes a primary barrier, whereas supportive institutional policies and strong professional identity mitigate constraints. These findings offer actionable insights for policymakers and educational leaders to reform compensation structures and workload management, thereby fostering more equitable career advancement for women in China.

## 1 Introduction

In recent decades, China’s rapid economic development and structural transformation have triggered profound changes in its social stratification system. The question of how individuals achieve upward social mobility—the movement from lower to higher socioeconomic positions—has become central to understanding inequality, opportunity, and modern labor dynamics (Zhou, 2000). Among the most critically affected groups in this transformation are Chinese women, who must navigate not only the structural challenges of an evolving labor market but also deep-rooted gender norms and family responsibilities. Investigating how women perceive and pursue upward mobility in contemporary China is essential for improving gender equality, ensuring inclusive development, and addressing systemic barriers that constrain professional advancement.

Upward social mobility has long been seen as a crucial mechanism for individuals to achieve class advancement and for societies to promote equity (Tencent News, 2024). However, existing research shows that access to upward mobility is not equally available to all groups. For women in the workplace in particular, the paths to mobility are often more complex and constrained. The current literature generally falls into the following three categories:

The first group of studies approaches the issue from a gender perspective. These works argue that women are often compelled to conceal their class backgrounds in professional settings due to their gender identity. This suppression of the “authentic self” contributes to experiences of marginalization and self-negation within organizations, sometimes leading to patterns of self-exclusion or voluntary withdrawal from upward mobility opportunities (Friedman, 2022). The second group focuses on class-based dynamics. Even when women succeed in moving upward, they often remain symbolically constrained by their origins. For instance, the lasting effects of territorial stigma continue to shape how they are perceived and treated, making it difficult to fully escape the disadvantages tied to their class background—even in elite professions (Born, 2023). Similarly, women from rural or marginalized ethnic backgrounds may obtain access to higher education, yet continue to face significant cultural and institutional barriers both within and outside university settings (Sepúlveda and Lizama-Loyola, 2022). The third strand of research highlights biases rooted in specific industries or cultural settings. These studies show how aesthetic norms can serve as implicit but powerful gatekeeping mechanisms. For example, Black women in the workplace often encounter a tension between professional advancement and appearance-related expectations—such as bias against natural hair—revealing how external image becomes an invisible standard that can impede career progression (Summers et al., 2022).

Although these studies offer valuable theoretical and empirical insights into the barriers women face in the process of upward mobility, three key limitations remain.

First, current research tends to oscillate between structural determinism and agency-centered identity frameworks. On one hand, studies emphasizing class labeling or territorial stigma highlight structural constraints but often overlook how women actively negotiate, reinterpret, or resist these constraints. On the other hand, work focused on self-presentation or aesthetic discipline highlights women’s agency but tends to underplay the influence of broader institutional and policy environments on individual choices. As such, there is an urgent need for a meso-level theoretical approach that bridges the macro–micro divide, integrating both structural forces and individual agency.

Second, most existing studies rely on qualitative interviews or are situated in specific national contexts—such as the UK, Germany, Norway, or the United States—lacking systematic evaluations of the relative weight of different influencing factors. This makes it difficult to identify which variables are most critical in constraining women’s career mobility and limits the practical guidance such research can offer for policy prioritization and intervention.

Finally, the majority of research centers on immigrant women, Black women, or women from lower-class backgrounds in Western contexts. There remains a clear gap in attention to women in China, particularly those navigating the dual pressures of “involution” and gendered anxieties during a period of rapid social transformation. Ordinary educated women in China remain underrepresented in the literature. Therefore, there is a pressing need for more localized empirical research that incorporates China’s unique institutional structures and cultural conditions, and adopts a framework that meaningfully integrates structural constraints with individual agency.

To address these gaps, this study investigates how Chinese women articulate their experiences, constraints, and aspirations related to upward social mobility. Drawing on 4,043 public comments by self-identified female users on Zhihu, one of China’s largest knowledge-sharing platforms, we apply Latent Dirichlet Allocation (LDA) topic modeling to extract key thematic patterns. Anchored in social comparison theory and supplemented by the framework of structuration theory, this study asks: What structural and individual-level factors influence how Chinese women perceive and pursue upward social mobility? Through this analytical lens, we aim to uncover the mechanisms by which women understand and negotiate their career advancement within the context of China’s evolving socioeconomic structure.

This research offers several contributions. Theoretically, it enriches the social comparison theory by demonstrating how Chinese women’s evaluations of self-worth, opportunity, and mobility are shaped by both structural inequalities and peer-based comparisons. Methodologically, it showcases the utility of computational text analysis for studying large-scale digital discourse, allowing for a more nuanced understanding of women’s mobility perceptions across social strata. Empirically, the study highlights key dimensions such as economic reward, time-energy constraints, education, gender bias, and regional opportunity as central to women’s perceived mobility paths. By focusing on organically expressed experiences and comparative narratives, this study provides a fresh, bottom-up perspective on gendered social mobility in contemporary China.

## 2 Theoretical analysis and research hypotheses

A review of existing research indicates that the academic community has not yet provided a clear definition of “upward mobility.” Nevertheless, behaviors associated with “upward mobility” are increasingly attracting attention among contemporary Chinese youth. Within the context of workplace upward mobility, individuals with higher educational qualifications tend to have greater opportunities for career advancement (El-Mallakh and Wahba, 2021; Kidd, 2012). Individuals with higher education levels are often driven by favorable career environments and higher financial incentives, leading them to pursue employment in occupations of mid- to high-prestige, where opportunities for upward mobility are significantly greater (Huang et al., 2025)Chinese mid- and high-level professionals typically possess stronger social capital and greater economic resilience, enabling them to frequently move across regions and make significant career advancements. Consequently, a large portion of the Chinese workforce actively pursues higher social status, further intensifying workplace competition.

Festinger’s Social Comparison Theory (1954) proposes that individuals achieve clear self-evaluation by comparing their own situation with that of others. Through these comparisons, individuals form relatively stable perceptions of themselves (Festinger, 1954). Social comparisons can be categorized into three types based on their direction: upward, downward, and lateral comparisons. Upward social comparison occurs when individuals compare themselves with others who are perceived as superior. According to Festinger, this type of comparison helps individuals clearly assess their own abilities and achievements. Wheeler and Miyake (1992) further elaborated that upward social comparison often leads individuals to internalize feelings associated with others’ achievements or failures, anticipating similar outcomes for themselves. (Tesser, 2000) highlighted that upward social comparisons could provoke negative emotions, such as envy or frustration (Vecchio, 2005), when individuals perceive others as outperforming them (Tesser, 2000; Van Loo et al., 2013).

This study hypothesizes that the intensity of social comparison directly influences individuals’ willingness and capacity to achieve upward mobility. To empirically examine this, comments from Chinese female users on the Zhihu platform, under the topic of “involution,” were analyzed using the Latent Dirichlet Allocation (LDA) model. This approach objectively captures the key themes and concerns related to workplace competition and upward social mobility among Chinese women. By exploring these comments, this study aims to provide novel insights into the challenges and constraints faced by women striving for career advancement in contemporary China.”

### 2.1 Negative paths to upward social mobility

The rewards received by coworkers with the same qualifications are an important source of comparison for individuals in the workplace and can have a significant impact on them. According to social comparison theory (Festinger, 1954) and studies on how employees compare their returns to work with those of colleagues (Luna-Arocas and Tang, 2024; Pillinger, 2023), horizontal pay comparisons have become a central way for individuals to evaluate fairness within organizations.

When women in the workplace observe pay differences among colleagues with similar qualifications, these direct comparisons often raise concerns about distributive justice (Luna-Arocas and Tang, 2024). In the Chinese labor market, such concerns are particularly salient given the persistence of traditional gender role expectations and structural inequalities across employment sectors. Many women, despite holding similar educational backgrounds and professional responsibilities as their male colleagues, find themselves placed in lower-paying roles or excluded from key performance-based bonuses and promotion channels. This phenomenon is often reinforced by implicit organizational practices that reward male-dominated positions with higher visibility and evaluation advantages. As a result, women are more likely to experience stagnation in career advancement and to perceive a lack of institutional recognition for their contributions (Ma, 2025).

This unequal distribution creates a double challenge. On one hand, the feeling of relative deprivation caused by pay comparisons undermines the perception of fairness (Li, 2022). On the other hand, the positive relationship between perceived organizational fairness and job performance is weakened (Faeq and Ismael, 2022). As a result, a harmful cycle may emerge: perceived unfairness leads to lower motivation, which in turn leads to reduced performance.

*H1*: The pay gap is negatively related to upward social mobility

Women in the workplace often face the challenge of time poverty, which stems from the ongoing struggle to balance professional and family responsibilities. In China, this issue is particularly pronounced due to the strong societal expectations placed on women to fulfill caregiving roles within the household. As work hours become increasingly fragmented and competitive pressures intensify in urban labor markets, many women find themselves juggling extended work commitments alongside responsibilities for child-rearing and elder care (Lu, 2024).

Although China has made significant strides in expanding educational and employment opportunities for women, the structural disadvantages they face in both the labor market and the family domain remain persistent. Women are more likely to be employed in lower-paid, less secure jobs, and simultaneously bear a disproportionate share of unpaid domestic labor (Clawson and Gerstel, 2014; Türkeli et al., 2023; Gadzali and Ausat, 2023). As a result, their career trajectories are frequently characterized by discontinuity and compromise. While the professional environment increasingly rewards long working hours and geographic mobility, many women must forgo such opportunities due to caregiving responsibilities, creating a fragmented pattern of career development similar to that observed in other contexts (Acker, 2006).

For Chinese women who have entered motherhood, the challenges often intensify. Balancing early child care with full-time employment can lead to role conflict, emotional strain, and a perceived lack of advancement opportunities. In some cases, the incompatibility between institutional expectations and family obligations prompts women to temporarily or permanently withdraw from the workforce (Sabellis and Schilling, 2013; Hirschman, 1970). These sacrifices—often unacknowledged in organizational structures—underscore the extent to which professional success for women still demands significant personal compromise (Bolton and Muzio, 2008).

Despite repeated efforts to legislate gender equality in the workplace, practical enforcement remains uneven. Reports continue to show that many women experience implicit discrimination (Triana et al., 2019), limited promotion pathways, and even harassment (Kim et al., 2020), further dampening their motivation to pursue upward mobility (Webber, 2019). Based on these conditions, this paper hypothesizes that when women are subjected to the dual pressures of work and family life, their motivation for career advancement tends to decline—thereby reinforcing the cycle of gender inequality.

*H2*: The “time-energy deficit” dilemma of working women hinders career development.

Women’s career expectations are significantly shaped by gender differences, and in China, this influence is deeply embedded in the sociocultural and institutional fabric of the labor market. Chinese women often interpret their workplace experiences through a gendered lens, particularly in sectors where male dominance is structurally entrenched and organizational hierarchies reflect long-standing gender norms (Shymko et al., 2024). Although achievement motivation exists across genders, the relationship between aspiration and career advancement tends to differ, with women exhibiting more cautious or restrained expectations under the influence of workplace realities (Mendez and Crawford, 2002; Guoying, 2011).

Through processes of social comparison, many Chinese women perceive greater difficulty in accessing promotions or leadership roles compared to their male peers (Watts et al., 2015; Zhang and Basha, 2023). These perceptions are not unfounded; they are continuously reinforced by implicit and explicit gender stereotypes that inform hiring decisions, task assignments, and promotion standards (Wood, 2008; Heilman, 2012). Under such circumstances, women may internalize these constraints, leading to reduced self-confidence and lower expectations regarding their own career trajectories, especially in positions requiring assertiveness, visibility, or external networking.

Such internalized barriers are further exacerbated by limited access to resources that could counterbalance structural disadvantages. For instance, the narrow range of career strategies deemed socially acceptable for female managers in China limits their ability to build effective social capital (Zhu et al., 2016). This not only restricts upward mobility but also reinforces occupational segregation in many industries. Moreover, studies suggest that only a small proportion of women perceive themselves as having the same potential to attain senior positions as their male counterparts, highlighting a self-assessment gap that is shaped by organizational culture and institutional expectations (Elliott and Smith, 2004).

Gender-based disparities in career expectation formation are often rooted in early experiences and pre-employment socialization. Inequities in the labor market manifest even before career entry, as gendered expectations guide educational choices and initial job aspirations (Schweitzer et al., 2011). In the Chinese context, differences in age, education level, and income intersect with cultural norms to produce distinct labor market experiences and anticipations for men and women. Traditional gender stereotypes continue to serve as templates for defining professional roles, shaping how women see themselves within the broader career structure (Wei, 2011).

Based on this evidence, this paper proposes that gender plays a decisive role in shaping expectations for upward social mobility in China—not only through institutional practices and external constraints but also through the internalization of role expectations and societal norms.

*H3*: Gender bias is negatively related to career expectation gains.

### 2.2 Positive paths to upward social mobility

According to social comparison theory, individuals engage in horizontal comparisons in the workplace to evaluate their relative standing within a group (Festinger, 1954). In the Chinese context, such comparisons often center on educational credentials, which serve not only as indicators of professional competence but also as key filters in institutional selection processes. Many professionals assess their own competitiveness by comparing their degrees, university backgrounds, and academic achievements with those of their colleagues, especially in sectors where formal qualifications are rigidly standardized.

Educational attainment continues to play a critical role in hiring and promotion within China’s increasingly credential-driven labor market. As observed in broader comparative studies, higher education may lead to greater job autonomy for women (Wu et al., 2021), and a strong academic background often serves as a proxy for productivity and potential (Habermalz, 2003). These dynamics are also evident in China, where elite universities and advanced degrees are often prerequisites for entry into high-paying or high-status roles, especially in public institutions and state-owned enterprises.

However, educational credentials alone do not guarantee upward mobility. In practice, the value of education is shaped by its alignment with institutional expectations, social capital, and family background. Some research has found that hiring for top- and upper-middle-level positions tends to favor candidates from economically privileged or elite backgrounds (Brieba and Herrera-Marín, 2025), a pattern increasingly visible in China’s urban job markets. Meanwhile, individuals from rural areas or with less prestigious degrees may find themselves excluded from career advancement opportunities, regardless of their actual ability.

These findings suggest that education level remains a powerful but selective mechanism for workplace mobility (Ropero-García, 2023). It not only influences how individuals compare themselves to others, but also determines their access to opportunity structures shaped by class, region, and institutional bias. In this way, educational attainment contributes both to the expansion and the reproduction of occupational hierarchies (Volkers et al., 2007).

*H4*: There is a positive relationship between education level and upward mobility in the workplace.

Income levels and cost of living are both key factors influencing regional economic development. While there is a clear correlation between income and the cost of living, studies have found that both variables tend to increase with city size (Campbell and James, 2020). In the Chinese context, this pattern is especially prominent in first- and second-tier cities, where higher per capita incomes are often accompanied by significantly elevated living costs. Nonetheless, these urban centers remain attractive to job seekers due to the wider range of employment opportunities and more robust social infrastructure they offer (Patacchini and Zenou, 2006). As David Rusk once noted, “The worst neighborhoods ruin good plans.” In rapidly urbanizing China, major metropolitan areas such as Beijing, Shanghai, and Shenzhen are typically better equipped to support income growth, provide diverse job opportunities, and deliver essential services such as housing and healthcare, further enhancing their appeal to the labor force (Squires and Kubrin, 2005).

Moreover, research shows that individuals’ career aspirations are strongly shaped by the opportunity structures embedded in urban environments. In China, where access to quality education, stable employment, and public services is highly stratified across regions, limited opportunities in smaller or less-developed cities often motivate individuals to strive for upward mobility through geographic relocation or intensified personal effort (Furlong et al., 1996). Empirical studies support the idea that upward mobility is more likely to occur in Chinese cities that are larger in population, well-connected by transportation, and characterized by lower income inequality and higher levels of educational attainment (Michelangeli and Türk, 2021). Furthermore, domestic research has demonstrated a positive relationship between upward mobility and local economic conditions such as high GDP growth and low unemployment rates, underscoring the importance of economic prosperity in shaping mobility outcomes and maintaining social stability in China (Chen et al., 2018).

*H5*: The scale of urban development is positively associated with upward social mobility.

## 3 Research design

### 3.1 Data collection and corpus construction

This study examines Chinese women’s perceptions of upward social mobility by analyzing user-generated content from Zhihu, a leading knowledge-sharing platform in China. Comments were collected from the “Workplace” and “Career Development” sections, focusing on threads in which women discussed career challenges and professional aspirations. Data collection was conducted using Octopus, a domestically developed web-scraping software that enables automated extraction and de-duplication of platform content. ^1^The initial dataset covers the period from January 1, 2022, to June 1, 2024, yielding a total of 4,043 raw comments.

To ensure sample relevance and reduce noise, several screening criteria were applied: (1) comments had to be authored by self-identified female users, as indicated by usernames, bios, or thread tags; (2) content had to pertain to career development, workplace experience, or mobility-related aspirations; (3) duplicate, irrelevant, or spam-like content was removed via keyword filtering and manual review. The final dataset included 4,043 valid comments.

It is important to note that Zhihu users tend to be younger, urban-based, and highly educated. While we did not explicitly control for occupation, age, or region due to data limitations, this demographic bias is acknowledged and discussed in the interpretation of results. Comments from students and early-career professionals were retained to reflect the platform’s core user group.

This research was approved by Fuzhou Yango University. All procedures involving data collection and analysis comply with the ethical standards and research integrity guidelines of the institution. As the data used in this study were publicly available on Zhihu, a Chinese knowledge-sharing platform, no personal or sensitive information was collected, and anonymity of users has been preserved throughout.

### 3.2 Data preprocessing

Before conducting topic modeling, we implemented a comprehensive preprocessing pipeline to ensure data quality and model validity (Zheng et al., 2015). The steps included:

1. Tokenization: We utilized the Jieba tokenizer with a custom dictionary, which included domain-specific terms such as “career ladder,” “glass ceiling,” and “promotion barrier.”
2. Stop-word Removal: A widely accepted stop-word list for Chinese language processing was used to eliminate high-frequency but semantically neutral terms.
3. Noise Cleaning: We removed punctuation, emojis, URLs, HTML tags, and other non-linguistic characters.
4. Synonym Merging: Frequently used variant terms were manually normalized to unify semantic categories.
5. Stemming and Lemmatization: Omitted due to Chinese’s lack of morphological inflection and the limited applicability of such steps.

### 3.3 LDA topic modeling and analysis

The study employs the Latent Dirichlet Allocation (LDA) model, a widely used unsupervised probabilistic model, to uncover latent thematic structures in the comment data. LDA assumes that each document (in this case, each comment) is composed of a mixture of topics, and each topic is characterized by a distribution over words.

LDA is a three-layer Bayesian model consisting of a document layer, a topic layer, and a word layer. Its core equation describes the conditional probability of a topic given a word:

$\begin{array}{l} P(c|o)=P(c|f_{i}|f_{j})=P(c|f_{i})\times P(c|f_{j})\\ =\frac{P(f_{i}|c)\times P(c)}{P(f_{i})}\times \frac{P(f_{j}|c)\times P(c)}{P(f_{j})} \end{array}$

In this context, let o represent the overarching issue discussed on Zhihu, and c denote specific subtopics or thematic categories. The term f refers to high-frequency words extracted from the discourse. Thus, P(c∣f)indicates the likelihood that a piece of text belongs to topic c when it contains the word f, while P(f∣c)represents the likelihood of word f appearing within texts categorized as c, and P(f)refers to the word’s overall frequency in the corpus.

#### 3.3.1 Determining the number of topics and model optimization

Determining the optimal number of topics (K) is a key step in Latent Dirichlet Allocation (LDA) modeling, directly affecting the model’s ability to extract meaningful themes. To identify the best value of K, we tested topic numbers ranging from 4 to 12, and evaluated each model using the perplexity score, a widely used metric for probabilistic models. Perplexity assesses how well the model generalizes to unseen data—lower perplexity indicates better performance.

**Figure 1:**
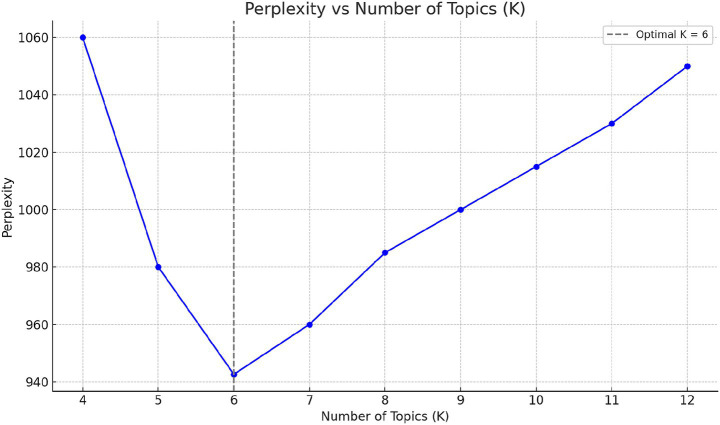
Perplexity curve with varying topic numbers.

As shown in Figure 1, the perplexity score decreased substantially from *K* = 4 to *K* = 6, reaching its minimum at *K* = 6 (perplexity = 942.6). Beyond this point, perplexity began to rise, indicating potential overfitting and reduced generalization capability. Based on this inflection point, we selected *K* = 6 as the optimal number of topics, as it provides the best balance between model simplicity and statistical performance.

Perplexity scores for different numbers of topics (Figure 1). The lowest perplexity (942.6) was observed at *K* = 6, indicating optimal generalization performance.

To enhance model robustness and interpretability, we conducted the following validation steps:

1. Multi-run stability check
  The LDA model was run 10 times with different random seeds. We computed the average Jaccard similarity across top-15 keywords in each topic between runs to evaluate the stability of topic-word distributions. The average similarity score exceeded 0.85, suggesting consistent topic structure.
2. Hyperparameter sensitivity test
  We tuned the Dirichlet priors *α* (document-topic distribution) and *β* (topic-word distribution) across several values:
  α ∈ {0.05, 0.1, 0.3, 0.5}.
  β ∈ {0.01, 0.05, 0.1}.
  For each combination, we recorded changes in both perplexity and topic coherence scores (using Gensim’s CoherenceModel with the C_V metric). The selected parameters α = 0.1 and β = 0.01 provided optimal trade-offs between generalizability and topic distinctiveness.
3. Topic coherence evaluation
  In addition to perplexity, we used topic coherence (C_V) as a semantic quality metric. The final model achieved an average coherence score of 0.49, which is within the acceptable range for short social media texts in Chinese.
4. Human interpretation reliability (coder validation)
  To ensure interpretability of the LDA outputs, we conducted manual topic labeling:
  Two independent coders, each with expertise in gender and labor studies in China, assigned descriptive labels based on top-20 words and 10 representative comments per topic.
  Inter-coder agreement was calculated using Cohen’s Kappa, yielding a high reliability coefficient of 0.87.
  Discrepancies in labeling were resolved through iterative discussion to ensure conceptual clarity and mutual understanding.
5. Visualization and interpretability check
  We used pyLDAvis to visually inspect each topic’s distribution across the corpus and assess topic separation. All six topics showed minimal overlap and well-separated semantic centers, reinforcing interpretability.

## 4 Findings

To evaluate the five hypotheses proposed in this study, we adopted a qualitative discourse analysis framework grounded in LDA topic modeling and network-based keyword co-occurrence. Rather than relying on formal statistical tests, each hypothesis was assessed based on the semantic consistency, keyword distribution, and recurrent comparative narratives in user-generated text. Specifically, a hypothesis was considered supported if:

High-frequency keywords aligned with the constructs defined in the hypothesis (e.g., salary, fairness, workload);

These keywords consistently co-occurred with relevant contextual terms (e.g., “salary” with “company,” “unfair,” or “resign”);

The surrounding comments expressed perceived injustice, constraint, or agency, consistent with the theoretical proposition;

Multiple, thematically consistent examples emerged across different topics or user groups.

Given that the data consists of naturalistic, unstructured text, we acknowledge that our evaluation approach is interpretive rather than inferential. The aim is not to quantify effect sizes or statistical significance, but to identify dominant experiential patterns and narrative mechanisms underpinning perceived mobility.

### 4.1 Results of data analysis

**Figure 2:**
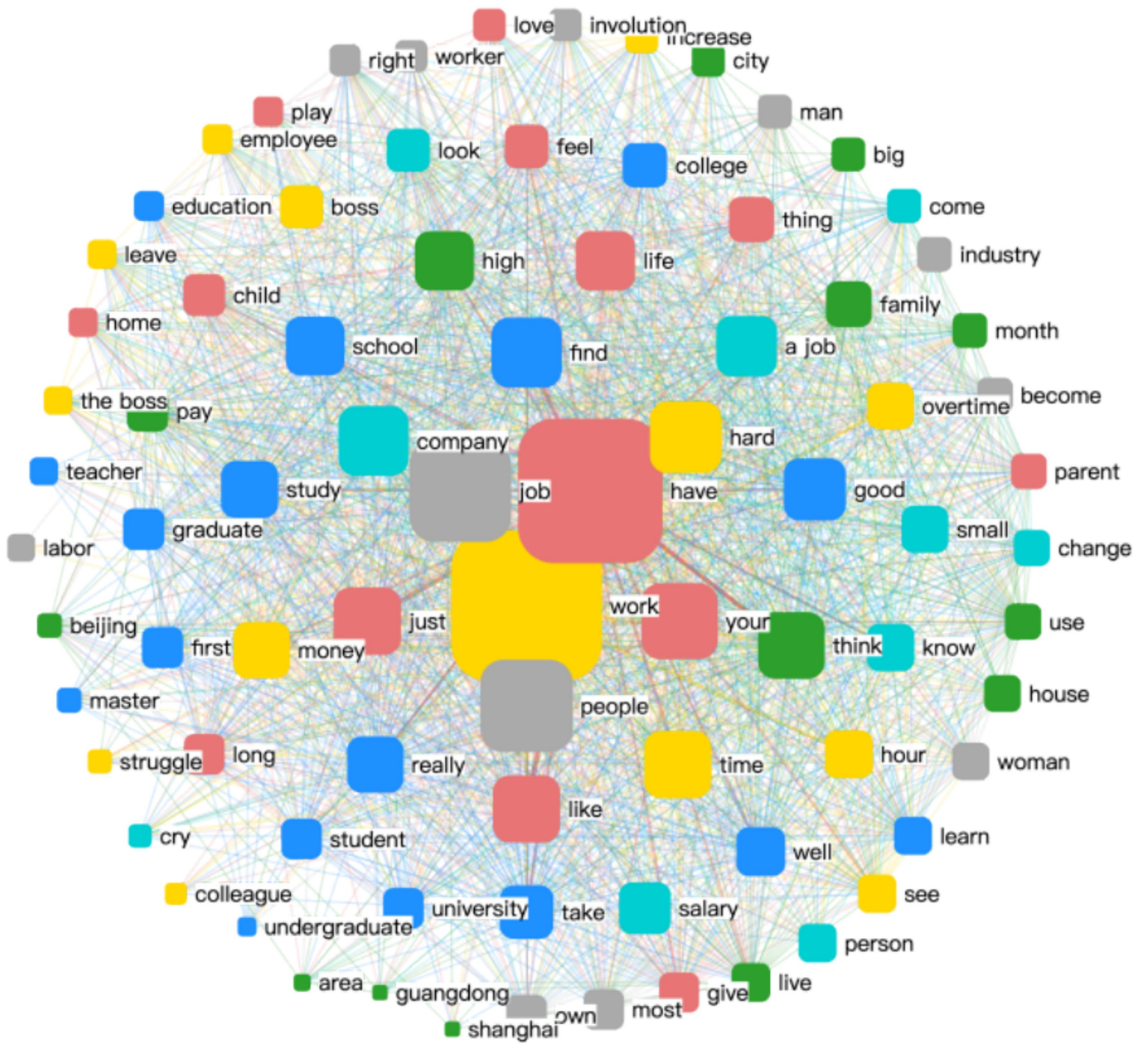
Illustrates the network relationships among the identified thematic categories.

A machine learning model was employed to extract topic words, and after parameter tuning, the optimal number of topic categories was determined by calculating perplexity. Based on this metric, the best semantic extraction was achieved when the number of topics was set to six. The results of the topic word extraction are illustrated in Figure 1, and the identified themes are as follows:

Yellow Theme: This theme reflects the multidimensional experience of the workplace and its core components. Centered around the keyword “work,” it is closely linked with terms such as “employee,” “increase,” “hard,” and “money,” highlighting the intensity and value-driven nature of professional life.
Blue Theme: This theme centers on educational background and its intrinsic connection to career development. High-frequency words include “college,” “study,” “education,” and “research,” indicating the foundational role of academic attainment in shaping professional trajectories.
Cyan Theme: This theme captures the multiple challenges and difficulties faced by women in the workplace. Key words such as “change,” “company,” “look,” and “salary” reflect concerns about organizational environment, job transitions, and pay disparities.
Red Theme: Focused on the impact of personal life on career development, this theme includes words like “life,” “love,” and “parent,” pointing to the ways in which family and emotional experiences intersect with professional paths.
Gray Theme: This theme addresses the nature of gender relations, particularly the dynamic between “men” and “women.” It reveals how gender continues to shape perceptions and interactions in professional contexts.
Green Theme: This theme emphasizes the influence of geographical factors on workplace development. It explores how spatial concepts such as “territory” interact with the notion of the “workplace,“highlighting regional differences in career opportunities and labor dynamics (Figure 2).

### 4.2 Interpretation of key textual themes through social comparison theory

#### 4.2.1 Comparison between workplace rewards and self-perception

Most Chinese women in the workplace tend to evaluate the balance between workplace rewards and their self-perceptions through comparative thinking, particularly when engaging with yellow and green topics. Within organizational settings, employee behavior is often shaped by their perceptions of fairness (Rutte and Messick, 1995). Consequently, Chinese women frequently compare their work conditions—such as salary and treatment—with those of their colleagues (Oldham et al., 1986). These comparisons can be divided into horizontal and vertical types. Horizontal comparison involves assessing one’s salary or benefits in relation to peers, especially colleagues in similar positions with comparable experience. Chinese women in the workplace often focus on individuals who share similar structural characteristics as the primary reference group (Shah, 1998).

**Figure 3:**
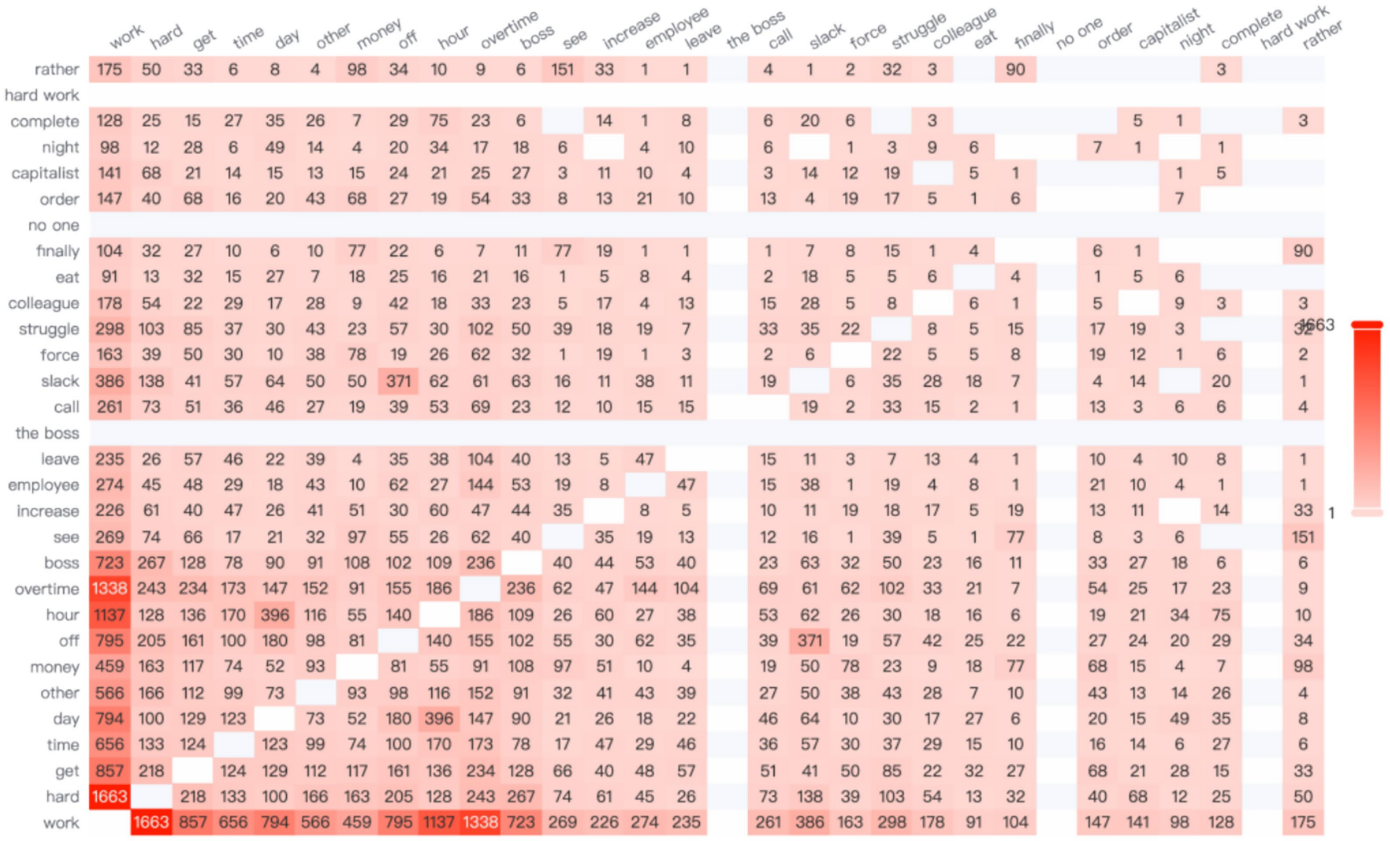
The network relationship co-occurrence diagram.

According to the data, the high-frequency word “salary” most frequently co-occurs with the word “company” (see Figure 3), highlighting the centrality of organizational compensation practices in such comparisons. In contemporary Chinese society, there is a widespread belief that high workplace rewards are directly associated with high pay. However, when female employees receive lower compensation than their peers—particularly male coworkers—they often experience a strong sense of unfairness.

“My colleague and I have the same education and years of experience, but his year-end bonus is twice as much as mine. The company never explained why.” (User xixi).

—This reflects a classic “unequal pay for equal work” situation and highlights women’s acute awareness of unfair treatment in compensation.

“Male coworkers around me always get assigned to the core projects. I work just as hard as they do, but their performance reviews are always better.” (User wenzi).

—This conveys frustration about hidden barriers to promotion and reveals how workplace hierarchies often favor men behind the scenes.

These perceived inequities intensify concerns surrounding the issue of “equal pay for equal work,” leading to increased anxiety and dissatisfaction. These negative emotions, in turn, may undermine motivation for upward social mobility. Based on this analysis, Hypothesis 1 is supported.

It is worth noting that Chinese women in the workplace also tend to make longitudinal comparisons—particularly based on company size—when making career choices. A co-occurrence network analysis of high-frequency words reveals that the term “small-company” appears with the highest frequency, ranking first, while the term “large-company” ranks fourth. This linguistic pattern aligns with empirical findings by Yousaf (2023), who used a dynamic panel data model to demonstrate that bankruptcy risk is significantly and positively correlated with profitability. The study found that small and medium-sized enterprises (SMEs) generally yield higher profits than large enterprises but also face a greater risk of bankruptcy (Yousaf, 2023).

This dual characteristic of “high return–high risk” may serve as a critical factor influencing how Chinese women assess the scale of potential employers. On the one hand, SMEs offer rapid promotion pathways and more flexible mechanisms; on the other, large enterprises provide more stable and structured career development. These contrasting features allow women with different career goals and risk tolerances to make differentiated employment decisions. By choosing organizational environments that better align with their personal and professional expectations, female employees may be able to reduce feelings of workplace burnout, particularly those arising from pay disparities.

#### 4.2.2 Comparison under limited time and scarce resources

When Chinese women in the workplace express concerns related to life, one of the central issues is how to rationally allocate time and resources. Cities with strong economic and educational infrastructures are highly attractive to Chinese talent, which has contributed to a “Matthew effect” in graduate employment—where advantages accumulate for those already in favorable positions. Currently, the prevalence of overwork, combined with insufficient rewards and resource scarcity, negatively affects employee engagement and well-being (Mazzetti et al., 2016).

**Figure 4:**
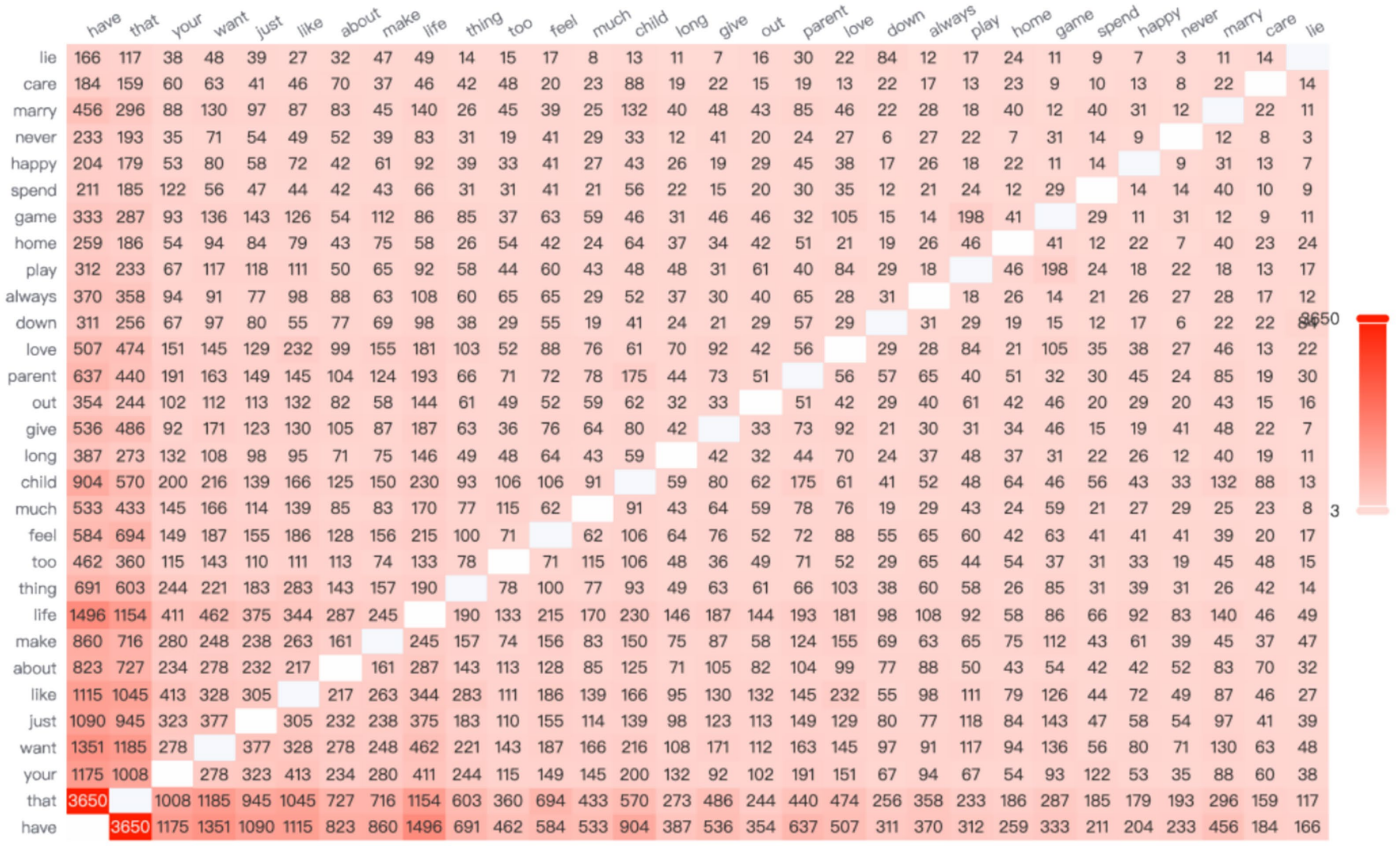
The network relationship co-occurrence diagram.

In the high-frequency word network analysis (Figure 4), the terms “have” and “life” show the highest co-occurrence, suggesting that women in the Chinese workplace have high expectations for maintaining a personal life despite professional demands. However, gender norms often restrict women’s autonomy over their time (Hyde et al., 2020). In the context of a gendered division of labor, women continue to carry a disproportionate share of unpaid domestic responsibilities (Deng, 2024). This structural condition of time poverty gradually transforms women’s desire to “have a free life” from a basic human need into a luxury or unattainable ideal.

“My whole day is just work and housework. I have not had a proper meal sitting down in weeks.” (User Angle).

—This comment reflects deep fatigue and loss of personal agency, caused by the pressure to juggle both career and family roles.

“Every time I see moms on social media who manage to work, cook, and still look good, I feel like I’m failing as a woman.” (User BB).

—This quote captures the emotional toll of social comparison and the guilt many working mothers internalize, even when their situations are objectively demanding.

Under conditions of high life pressure, Chinese women in the workplace often rely on social comparisons to evaluate whether they are successfully balancing work and life. In the context of time management comparisons, high-frequency words such as “love,” “right,” “play,” “feel,” “child,” “home,” “parent,” “life,” “cry,” and “like” suggest that beyond their professional roles, the emotional and personal aspects of life significantly shape how working women perceive their overall well-being.

Family relationships—such as those involving children (“child”), parents (“parent”), and intimate partners (“love”)—can lead to emotional fluctuations. The appearance of the word “cry” reflects the presence of negative emotions that may stem from the pressures of balancing life and work. In particular, women may feel stress or guilt when they compare themselves with others who seem able to dedicate sufficient time to both career and family. This type of comparison is especially pronounced among women of childbearing age.

Many mothers report experiencing guilt over their perceived inability to meet societal expectations surrounding childcare (Fielding-Singh and Cooper, 2023). As a result, a significant number of Chinese working women gradually reduce their workplace motivation when faced with the demands of multiple roles. Therefore, based on these observations, Hypothesis 2 is supported.

#### 4.2.3 Comparison under gender differentiation in the workplace

**Figure 5:**
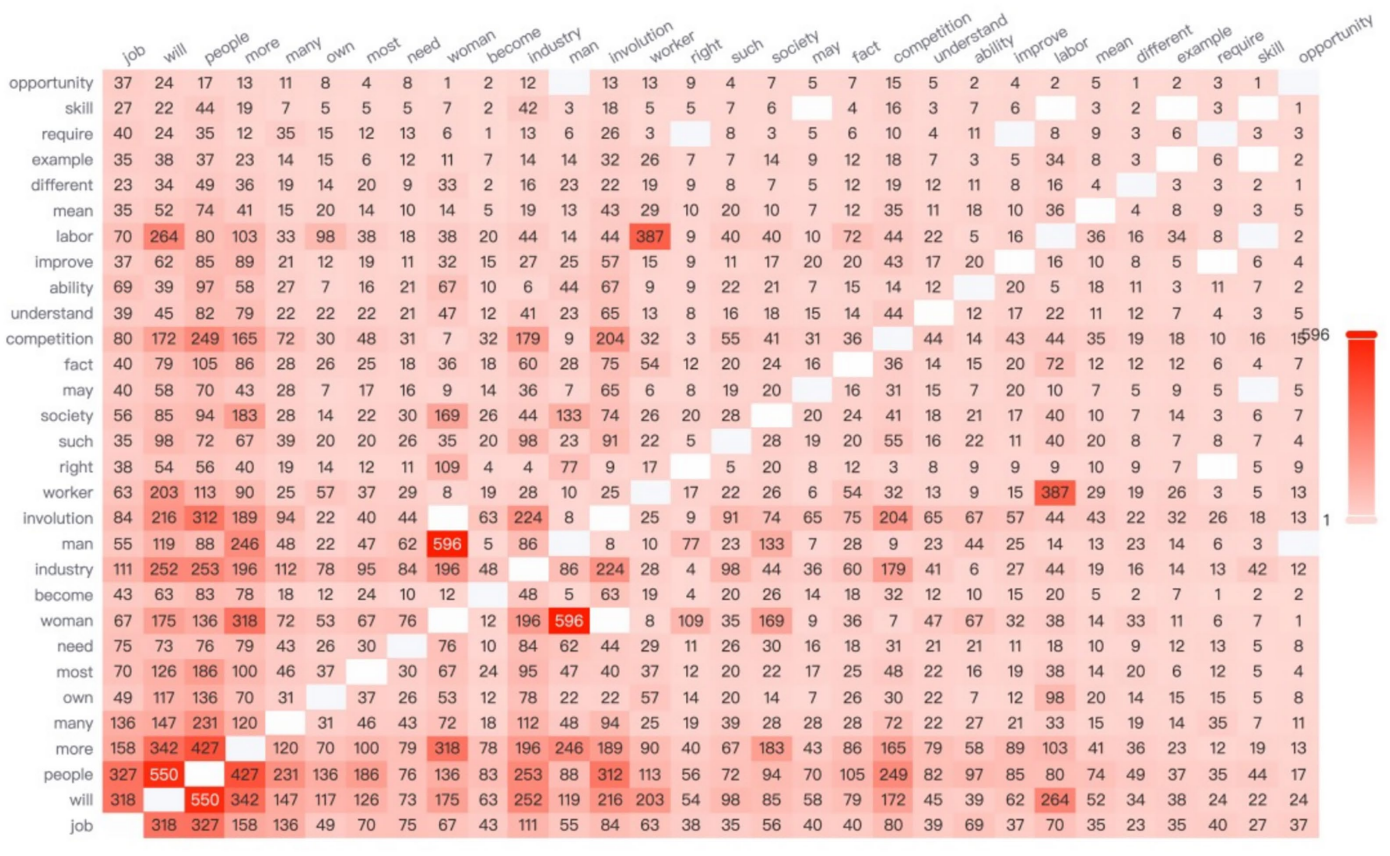
The network relationships of the impact of interpersonal gender communication.

When exploring the theme of interpersonal gender communication, Chinese women in the workplace often reflect on their experiences through the lens of social comparison, particularly in relation to gender roles. Based on the data analysis, there is a notably high co-occurrence between the words “woman” and “man.” As illustrated in Figure 5, the correlation frequency between these two terms reaches 596, indicating a strong semantic association. This linguistic pattern reflects broader concerns regarding gender bias in the workplace, especially in how women assess their work environments and overall job satisfaction.

“Whenever there’s a leadership role to fill, they say I’m ‘not aggressive enough,’ but my male colleague who barely speaks gets promoted.” (User E).

—This comment reveals frustration over gendered performance expectations and the invisibility of women’s qualifications in promotion decisions.

“Our team lead keeps saying men are naturally better at handling pressure. It’s like no matter how much we do, we are always seen as weaker.” (User F).

—This illustrates internalized gender stereotypes within the workplace and how repeated exposure to biased evaluations erodes women’s confidence.

Research has shown that women often face greater challenges than men in similar roles (Janssen and Backes-Gellner, 2016). Women have lower career expectation gains due to these perceptions of gender differences in the workplace (Sevilla and Snodgrass Rangel, 2023).

**Figure 6:**
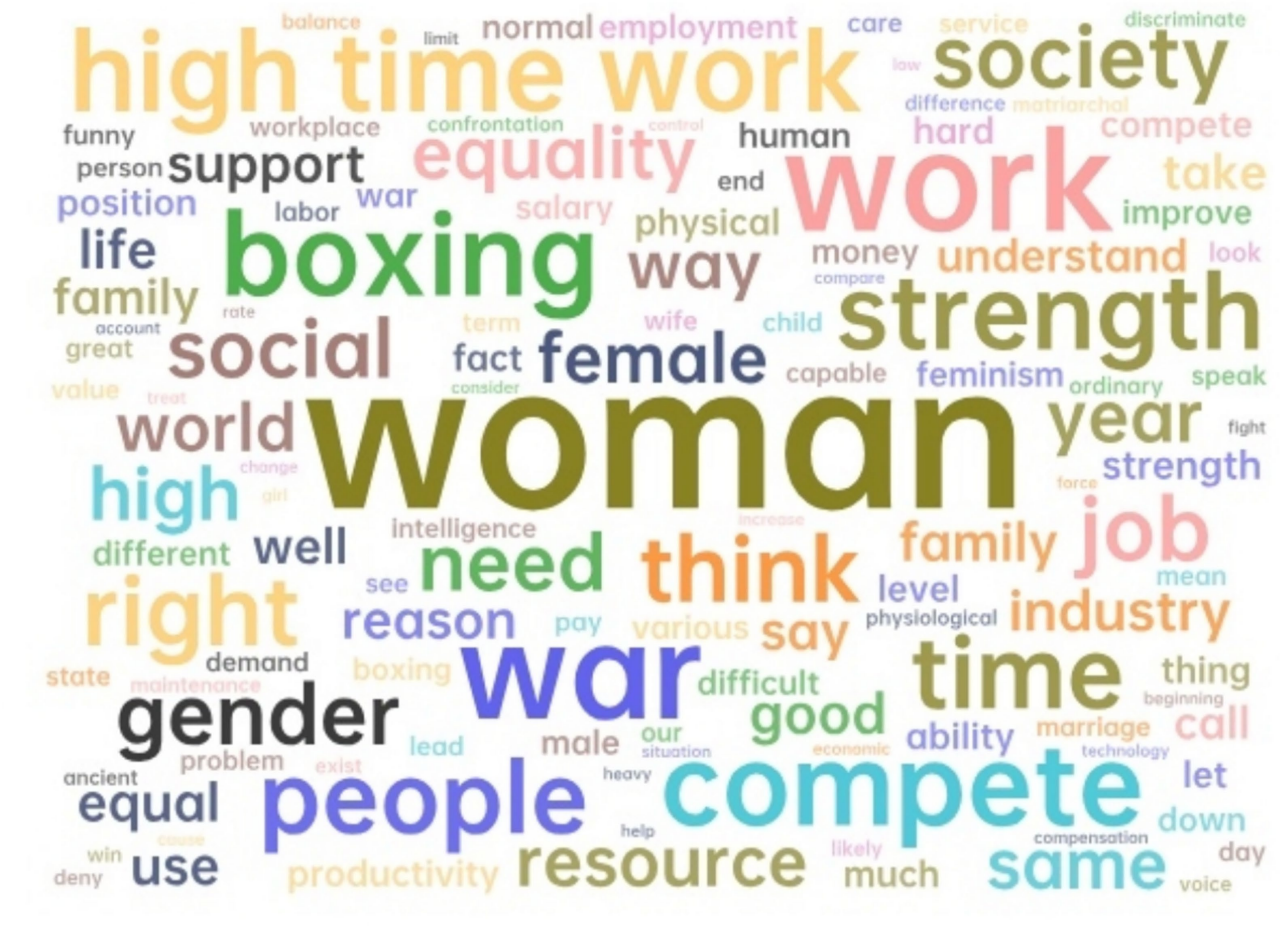
The network relationship diagram for “man”.

**Figure 7:**
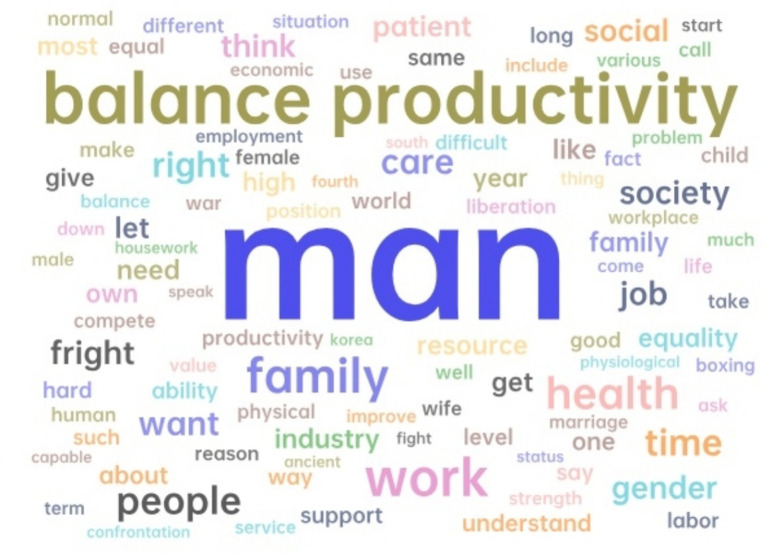
The network relationship diagram for “woman”.

To further study the content of women in the workplace discussing gender topics, the network relationship diagrams of “woman” and “man” were analyzed, as shown in Figures 6, 7. It can be further observed that the topics related to both genders differ significantly.

In the network map of “man,” keywords such as “war,” “boxing,” and “compete” form competitive imagery, mapping the narrative that male career progression is often constructed as a zero-sum game. The association between “high-time work” and “strength” implies that physical capital (e.g., hours of work, stress resistance) is used as a criterion for competitiveness assessment. Meanwhile, “support” and “family” are only marginal nodes, revealing a lack of social expectation for men to participate in caregiving.

In contrast, the “woman” network centers on “balance productivity,” with surrounding terms like “family,” “health,” and “care,” showing the enduring burden of domestic responsibility imposed on professional women. The appearance of emotionally conflicting expressions—such as “fight” versus “patient” and “let”—highlights the internal tensions women face between assertiveness and expected modesty. These findings indicate that women’s efforts to pursue career advancement are often accompanied by psychological and emotional costs not typically imposed on men (Lee and Wessel, 2022).

Thus, Hypothesis 3 is supported.

#### 4.2.4 Comparison under the education gap and the starting point of the workplace

Discussions under the theme of “education,” particularly around terms like “university,” “major,” “graduation,” and “graduate school,” illustrate how Chinese women evaluate their career starting points, capabilities, and future prospects through social comparisons during their transition from education to the workplace. Chinese women frequently engage in both horizontal and vertical comparisons to determine their career paths.

**Figure 8:**
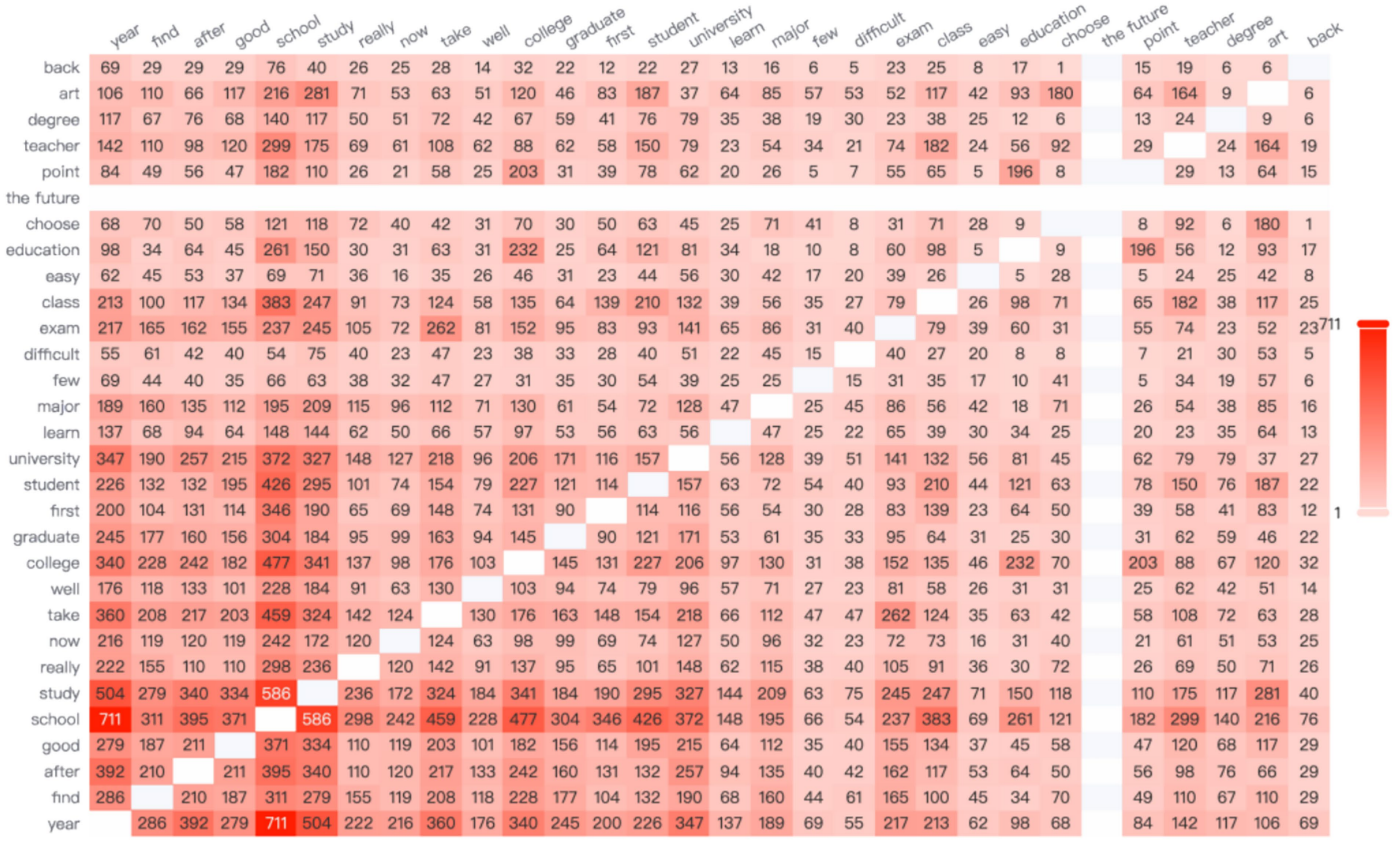
The network relationships under the theme of education.

In terms of vertical comparisons, age is a critical factor. In Figure 8, the terms “school” and “year” show the highest frequency of co-occurrence, indicating that Chinese working women often reflect upon their academic achievements and experiences at different ages. Rising inequality and transformations within higher education systems influence the career opportunities accessible to young people across different genders and socioeconomic backgrounds, shaping their perceptions and attitudes toward adulthood (Cepa and Furstenberg, 2021).

“If I had started my master’s degree two years earlier, I’d already be working in a top-tier firm now. Timing matters more than I thought.” (User GG).

—This comment reflects regret and anxiety over educational timing and its perceived long-term effects on career outcomes.

“Everyone in my team graduated from 985 universities. I feel like I’m always one step behind just because my school is not ‘famous enough.’” (User sad).

—This reveals internalized academic inferiority and the strong credential-based hierarchy in Chinese workplaces.

Chinese women in the workplace often engage in horizontal social comparisons, particularly regarding the academic backgrounds of their peers. As shown in Figure 7, the high-frequency words “school” and “study” exhibit the second-highest co-occurrence frequency, indicating that women frequently compare their educational experiences when considering career development.

Research has demonstrated that university reputation significantly affects the salaries of Chinese undergraduates (Hartog et al., 2010). As a result, comparisons related to university ranking and chosen major are common. Academic qualifications are widely regarded as indicators of an individual’s capability and career potential. If a woman does not attend a top-tier university or chooses a relatively less recognized major, she may develop feelings of inferiority and doubt about her future career prospects.

According to one study, among graduates with a master’s degree, those whose undergraduate degree is from a non-211 engineering institution are significantly less likely to receive resume responses than those from 211 engineering institutions, with a gap of up to 41% (Li and Bai, 2020). Therefore, Hypothesis 4 is supported.

#### 4.2.5 Geographical opportunities versus comparison under a limited economy

In analyzing this theme, it was observed that Chinese women frequently make social comparisons in workplace contexts across different geographical regions, emphasizing the contrasts between large and small cities and evaluating how regional career opportunities impact their professional advancement. Geographical differences thus emerge as a significant dimension of social comparison. Prominent cities such as Beijing, Shanghai, and Guangzhou were found to be focal points for discussion among Chinese working women, suggesting that superior job prospects and higher salaries positively influence their attitudes toward careers in first-tier cities (Jin et al., 2022).

“I moved to Beijing for a great job, but every month feels like a survival game—rent alone takes half my salary.” (User Iive).

—This comment captures the emotional exhaustion caused by urban cost pressure, despite access to better career opportunities.

“Without my parents helping with the down payment, I would not survive in a big city. I sometimes wonder if it’s worth the stress.” (User J).

—This reflects dependency on intergenerational support and doubts about whether geographic upward mobility is sustainable.

Additionally, economic development and future potential of a city significantly influence Chinese women’s career-related decisions. As shown in the network relationship diagram, the highest co-occurrence frequency is between the words “Beijing” and “house,” appearing 588 times (see Figure 8). This phenomenon directly reflects the central role of housing and survival pressures in China’s first-tier cities. It highlights a key issue in the development of women in the workplace: high housing prices create a talent filtering effect through a “hidden economic exclusion mechanism.” In other words, elevated housing costs significantly restrict the employment opportunities of low-educated migrants (Zhou and Hui, 2022).

Furthermore, decisions related to housing affordability and relocation are shaped by a combination of factors. The cost of “settling down” is no longer viewed purely through an economic lens—it increasingly involves intergenerational support. For example, the proportion of Chinese parents providing financial assistance for their children to purchase homes has tripled in recent years (Yu, 2020). Under this pressure, some young Chinese—even when career advancement opportunities are available—choose to leave first-tier cities in favor of a less risky and more sustainable lifestyle.

For contemporary Chinese working women, decisions about where to work are influenced by a range of factors. When planning their career paths, they have moved beyond the traditional city-size-oriented evaluation and instead developed a multi-dimensional assessment system that includes affordability as a core consideration. Specifically, housing costs—as a key source of economic pressure—play a crucial role in shaping career choices. When housing prices in major cities exceed an individual’s financial capacity, women may adopt a “city downgrading” strategy, opting for lower-tier cities with better cost–benefit ratios.

**Figure 9:**
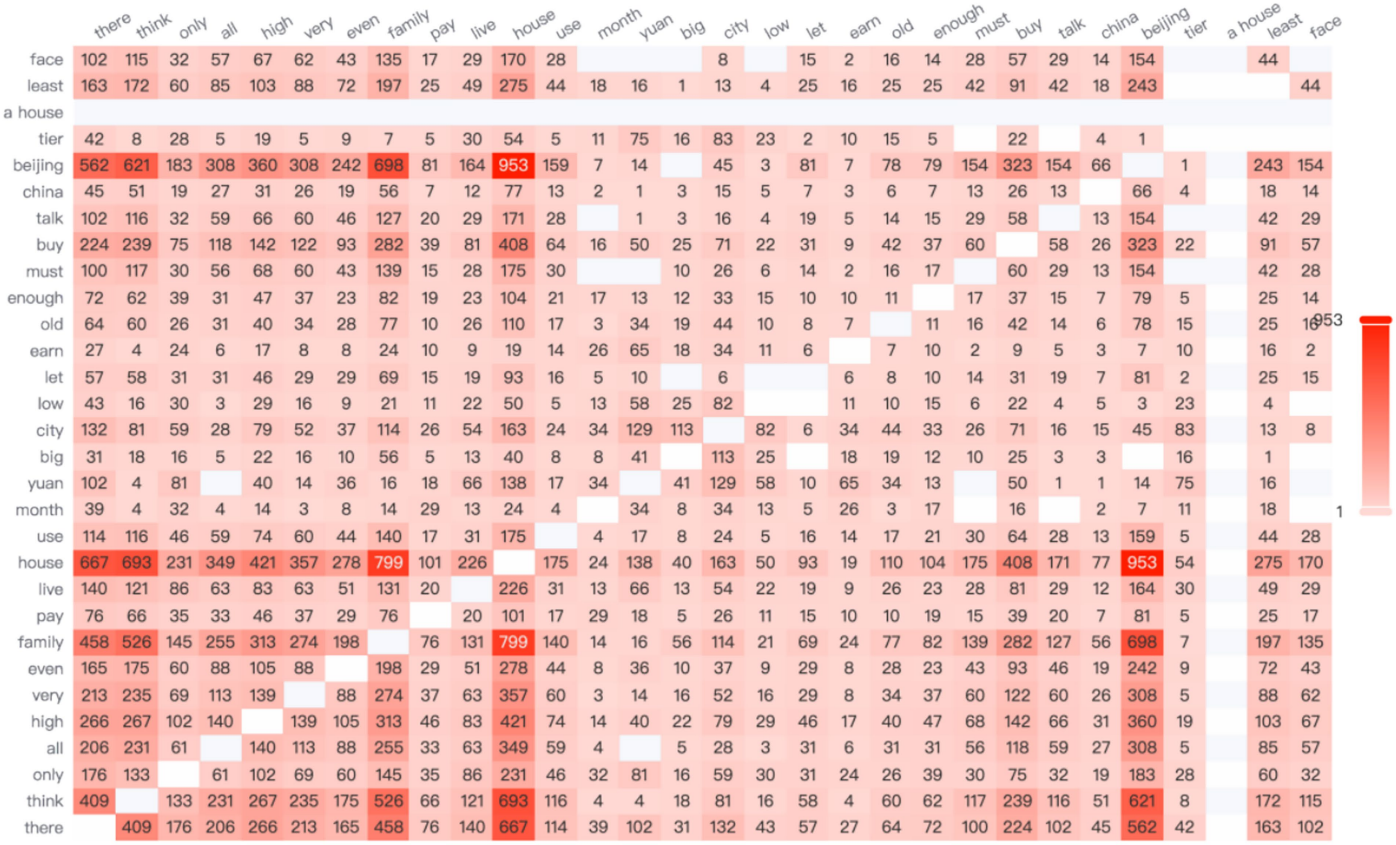
Illustrates the network relationships of geographic workplace mapping association domains.

Based on this, Hypothesis 5 is not supported (Figure 9).

## 5 Concluding remarks

### 5.1 Theoretical and practical implications for Chinese women’s upward mobility

Based on social comparison theory, this study explores the impact of upward social mobility by examining a representative case from the Chinese context. Specifically, the research analyzes 4,043 comments from Chinese women discussing workplace “involution” on the Zhihu platform, utilizing LDA topic modeling. The analysis reveals that Chinese women frequently engage in social comparisons when evaluating their career experiences, highlighting five critical dimensions that shape their perceived mobility: compensation and self-worth, educational opportunity, time-energy constraints, gender norms, and geographical barriers.

These findings contribute to the growing literature on gendered labor experiences by demonstrating how upward mobility is subjectively constructed through everyday discourse. Rather than treating institutional structures and personal agency as separate explanatory domains, this study integrates them through the lens of comparative reasoning, offering a more dynamic understanding of how women navigate professional inequality in contemporary China. In doing so, it advances the theoretical integration of social comparison and structuration frameworks while providing actionable insights for employers and policymakers seeking to create more equitable work environments.

Nevertheless, several limitations must be acknowledged. First, the data was collected from Zhihu between 2022 and 2024. As such, the findings reflect a specific temporal context and may not capture evolving sentiments in a rapidly shifting labor environment. Second, Zhihu users tend to be younger, urban-based, and more highly educated, leading to potential sampling bias. The discourse analyzed here thus represents a subset of China’s female labor force and may underrepresent the experiences of women from rural, older, or less-educated backgrounds. These demographic limitations should be considered when interpreting the generalizability of the results.

From a methodological perspective, while LDA modeling provides a scalable way to extract latent topics from large datasets, it has known limitations: it ignores word order, struggles with short texts, and is susceptible to overfitting. We mitigated these issues through rigorous preprocessing, topic validation, and multi-run stability checks. Still, future studies might consider using dynamic topic models or embedding-based methods (e.g., BERTopic) to improve interpretability and granularity.

In terms of future research, scholars could extend this study by incorporating multi-platform data to capture a broader demographic and more diverse narrative forms. Longitudinal analyses could also examine how women’s perceptions of upward mobility evolve across life stages or policy changes.

Beyond academia, our findings suggest several policy implications. Enterprises should consider conducting regular gender equity audits—assessing pay gaps, promotion rates, and workload allocation by gender—to detect and address structural biases. Governments and regional labor authorities could optimize talent retention policies by offering targeted housing support, childcare subsidies, or flexible work arrangements for professional women in second- and third-tier cities. Educational institutions may also revise career development programs to provide female students with early access to social capital and industry mentorship networks.

By recognizing these limitations and proposing actionable recommendations, this study serves as both a conceptual contribution and a practical reference for building more inclusive and responsive labor environments for women in China.

### 5.2 Contextualizing the findings: global comparison and China’s institutional specificity

The findings of this study reveal that the mechanisms shaping women’s upward mobility in the workplace reflect both globally shared dynamics and distinctive national characteristics. On one hand, many of the challenges faced by Chinese women—such as gender bias, unequal pay, and work–family conflict—are widely observed across global contexts, underscoring the persistence of structural gender inequality (van Doorn, 2023). On the other hand, these obstacles are filtered through China’s unique institutional arrangements and cultural expectations, resulting in localized expressions and pathways of constraint.

In Western contexts, researchers have long theorized concepts such as the glass ceiling to describe the invisible barriers women face in career advancement. These studies argue that gender stereotypes, motherhood penalties, and embedded structural biases within organizations collectively form a web of hidden exclusion (Espinosa and Ferreira, 2022; Purcell et al., 2010). The results of this study’s LDA topic modeling align closely with these findings. Many female users voiced frustration with being marginalized during promotions, receiving lower performance evaluations, and bearing disproportionate emotional labor—all of which resonate with the structural gender injustices identified in global scholarship.

However, unlike many Western countries where legal protections against discrimination are institutionally supported and broadly enforced, China presents a contrasting scenario. While formal gender equality is guaranteed under the law, enforcement mechanisms remain limited in scope and effectiveness (Sun, 2025). Discriminatory practices are often embedded in informal organizational norms and manifest covertly across hiring, evaluation, and advancement processes. Moreover, Chinese women’s career trajectories are strongly shaped by expectations tied to family ethics, intergenerational obligations, and collectivist cultural values—factors that are less prominent in more individualistic Western settings.

In addition to these legal and organizational disparities, Chinese women face a set of uniquely institutional and cultural constraints in their pursuit of upward mobility:

First, the hukou (household registration) system significantly restricts geographic mobility and reinforces regional inequalities. Women from rural or lower-tier urban backgrounds—even those with high educational attainment—often encounter institutional barriers that limit their long-term career prospects in top-tier cities (Hung, 2022).

Second, housing affordability functions as a form of hidden exclusion. Due to high real estate costs, many newcomers to large cities opt to rent rather than buy property (Mu et al., 2024). However, the financial burden of living in major cities such as Shanghai is widely reported among female professionals. Numerous users noted that without parental support for down payments, they would be unable to sustain a long-term life or career in these urban centers. This reflects the strong dependency on intergenerational financial support and reveals the extent to which professional geographic mobility is conditioned by familial wealth (Cui and Ronald, 2024).

Moreover, intensified competition within China’s educational system adds another layer of pressure. On one hand, the growing emphasis on elite credentials has made degrees from prestigious institutions a de facto requirement for desirable positions. On the other hand, women are frequently expected to perform multiple roles simultaneously—being a productive employee, a caring mother, and a socially acceptable woman—leading to what many describe as “total life management” burdens.

While structural impediments to women’s workplace mobility are certainly not unique to China, the interaction of the hukou system, real estate pressures, parental support expectations, credentialism, and deep-rooted gender norms combine to create a context-specific configuration of constrained mobility. This study thus not only confirms global findings on gendered inequality but also offers a structure–agency perspective that captures the particularities of Chinese women’s workplace experiences.

## Data Availability

The original contributions presented in the study are included in the article/Supplementary material, further inquiries can be directed to the corresponding author.

## Footnotes

^1^Octopus (also known as “八爪鱼” in Chinese) is a commercially available web-scraping software widely used in China. It enables automated collection of publicly available online content without requiring advanced coding skills. During the data collection process, the software complies with relevant legal regulations and privacy policies to ensure data security and legitimacy. For more information, see: https://service.bazhuayu.com/education/.
